# Supplementary material for: Implementing quality improvement intervention to improve intrapartum fetal heart rate monitoring during COVID-19 pandemic- observational study
Source: PLoS One. 2022 Oct 14;17(10):e0275801. doi: 10.1371/journal.pone.0275801 (PMC9565417; doi:10.1371/journal.pone.0275801)
Supplement: Attachment — Submitted filename: Response to reviewers comment.docx [file pone.0275801.s003.docx]

24 May 2022

Response to reviewer’s comment

Comment 1#. Why did you use median for time interval analysis? Had its normality been tested? Could you provide an explanation for this (if it's abnormal, then median was used,; if it's normal, then mean should have been used)

Response- The time interval from admission to abnormal fetal heart rate detection and time interval from abnormal FHR detection to time of delivery is not normally distributed. We did a normality test to respond to the reviewer’s query, please find below the time distribution using the histogram.

Comment 2#. Was the decreased interval from admission to abnormality detection decreased due to lack of human resources? It should have been presented in the discussion section.

Response- There are two possible reasons for decreased interval from admission to abnormality detection. First, is the lack of dedicated human resource to manage the high-risk mothers early on as the proportion of CS decreased as shown in table 5. Second, there is an increase in proportion of women admitted with complication at birth (5.5% before pandemic vs 12.5% during pandemic) as noted in table 3. We have added in the discussion section.

Comment 3#. Was the increased number of infants admitted to NICU during Covid 19 pandemic because of the good system or the pandemic itself or poor input of the infants? Please present it in the discussion section.

Response- The reasons for increase in number of infants admitted to NICU during pandemic despite improvement in early detection of abnormal fetal heart rate are same as above. First, the proportion of complication at admission increased due to increase in proportion of women with obstetric complication increased during pandemic. Second, the time to action despite early detection is lack of human resource to act upon women who require the care most. So, the pandemic has induced all two delays in care, delay in women coming to the health facilities and delay in high quality care.

Comment 4#. The conclusion reports the quality improvement, yet there was lack of human resources. Would you explain how many health workers the hospital should have had?

Response- We have now provided the estimated number of health workers the hospital should have to timely action on women who had abnormal FHR during labour.

Comment 5#. The article has generally been written in the standard English and is relatively easy for the readers to understand its content. There are, however, several or minor mistakes that have to be corrected. The mistakes are contained in both the Abstract and main body of the article. They include grammatical errors (inappropriate sentence structures), inconsistent use of the tenses (the result of a study is commonly written in the Simple Past Tense instead of the Simple Present Tense), and inappropriate use of adverbs and prepositions for some verbs. I hope the author will correct the errors prior to the publication.

Response- We have now done the copy edit.
